# Supplementary material for: Endosomal phosphatidylserine is critical for the YAP signalling pathway in proliferating cells
Source: Nat Commun. 2017 Nov 1;8:1246. doi: 10.1038/s41467-017-01255-3 (PMC5665887; doi:10.1038/s41467-017-01255-3)
Supplement: Supplementary file 1 — Supplementary Information [file 41467_2017_1255_MOESM1_ESM.pdf]

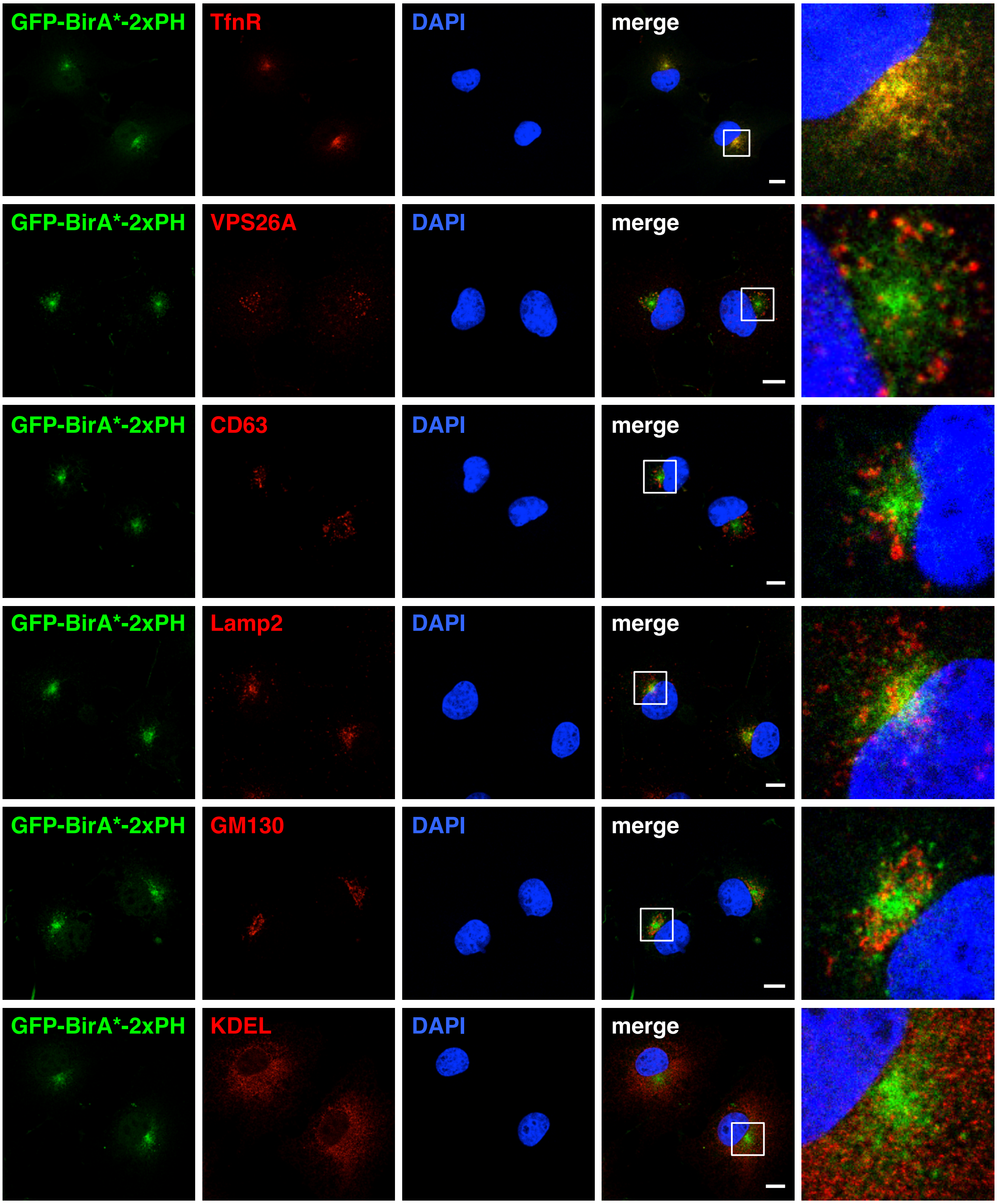

Supplementary Figure 1 RE localization of GFP-BirA*-2xPH. COS-1 cells that stably express GFP-BirA*-2xPH were fixed, permeabilized, and stained for TfnR (REs), VPS26A (early endosomes), CD63 (late endosomes), Lamp2 (lysosomes), GM130 (the Golgi), or KDEL (ER). Magnified images of the boxed areas around the perinuclear REs are shown in the right column. Nuclei were stained with DAPI. Scale bars, 10 µm.

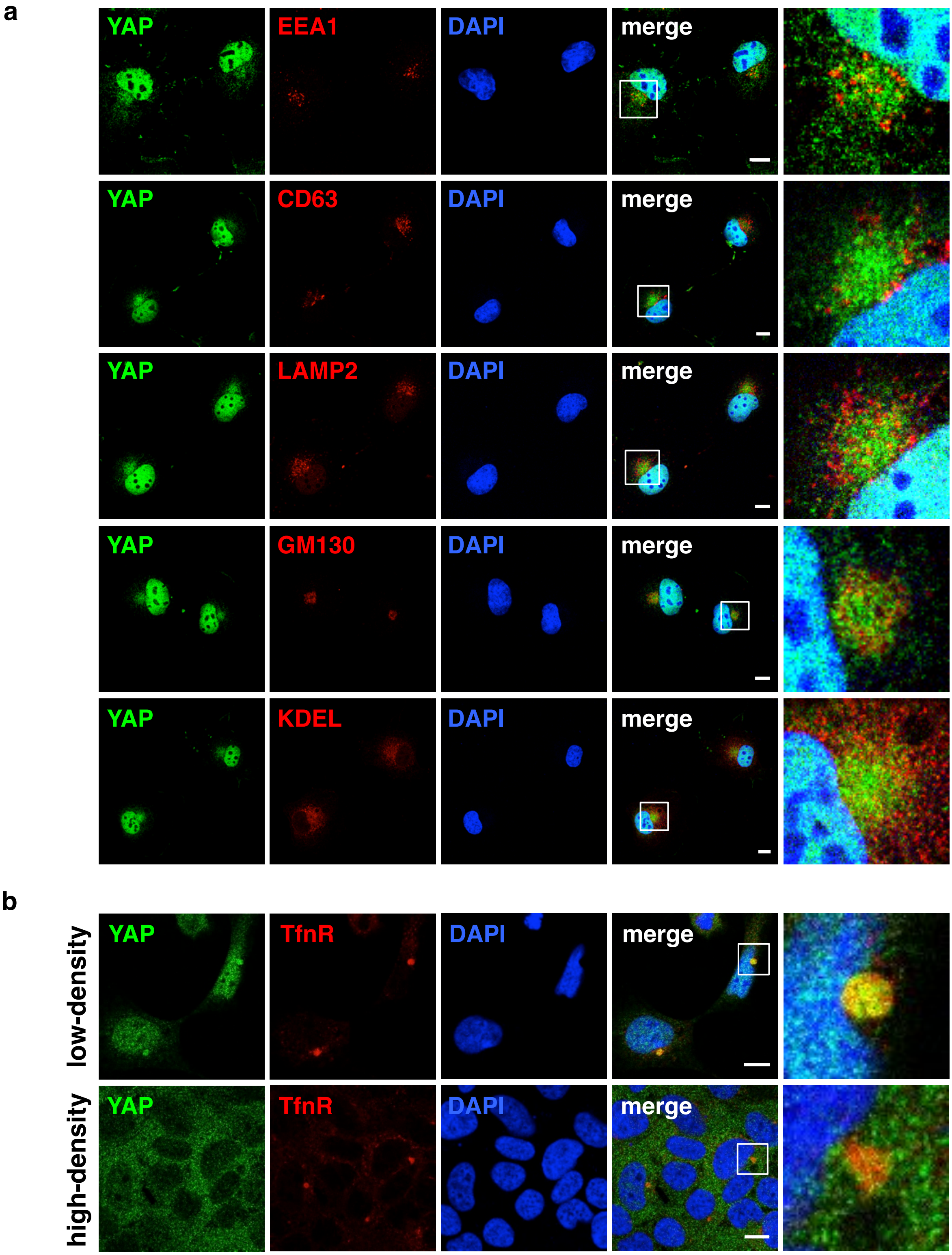

Supplementary Figure 2 RE localization of YAP in low-density COS-1 and HEK293A cells. (**a**) COS-1 cells were fixed, permeabilized, and co-stained for YAP and organelle markers: EEA1 (early endosomes), CD63 (late endosomes), Lamp2 (lysosomes), GM130 (the Golgi), or KDEL (ER). Magnified images of the boxed areas around the perinuclear REs are shown in the right column. (**b**) HEK293A cells at low or high density were fixed, permeabilized, and stained for YAP and TfnR. Magnified images of the boxed areas around the perinuclear REs are shown in the right column. Nuclei were stained with DAPI. Scale bars, 10 μm.

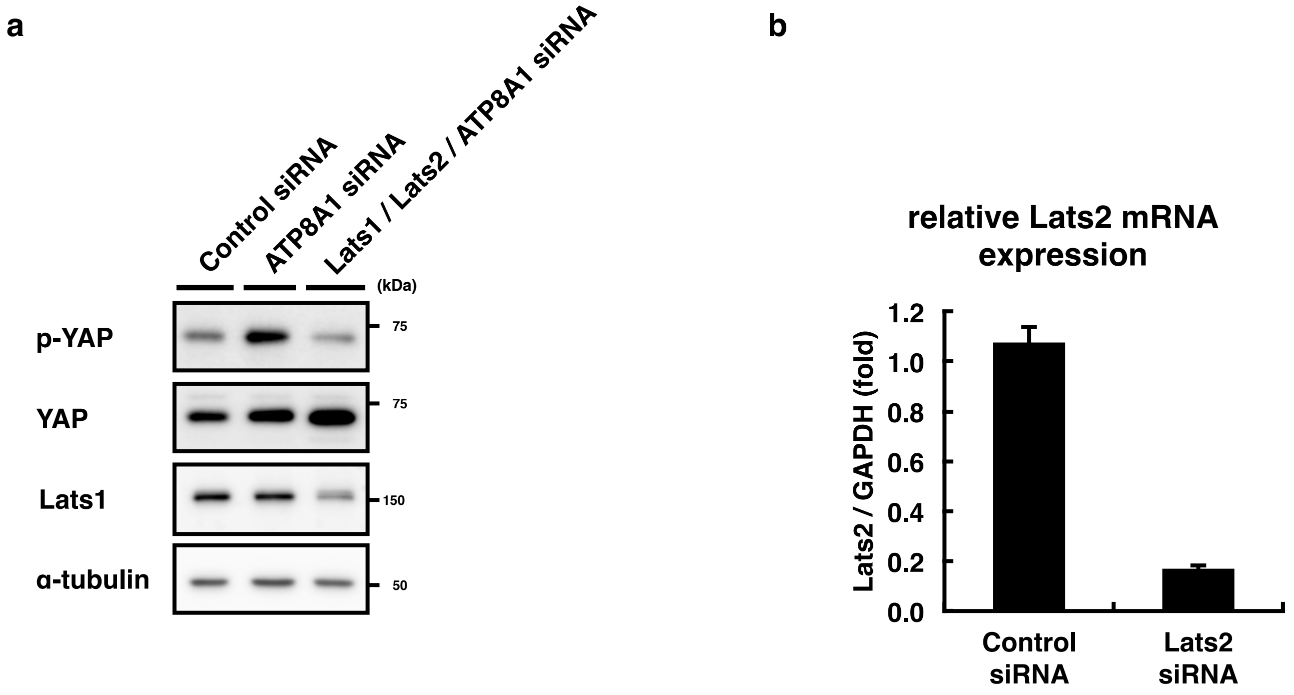

Supplementary Figure 3 The increase of phosphorylated YAP by ATP8A1 knockdown was reduced by depletion of Lats1/2. (**a**) COS-1 cells were treated with control, ATP8A1, or ATP8A1/Lats1/Lats2 siRNA for 48 h. Lysates of the cells were immunoblotted for YAP, p-YAP (S127), or Lats1. α-tubulin was used as a loading control. (**b**) qRT-PCR analysis of Lats2 mRNA from cells in **a**. GAPDH was used as an internal control. Data are mean ± s.d. for three independent experiments.

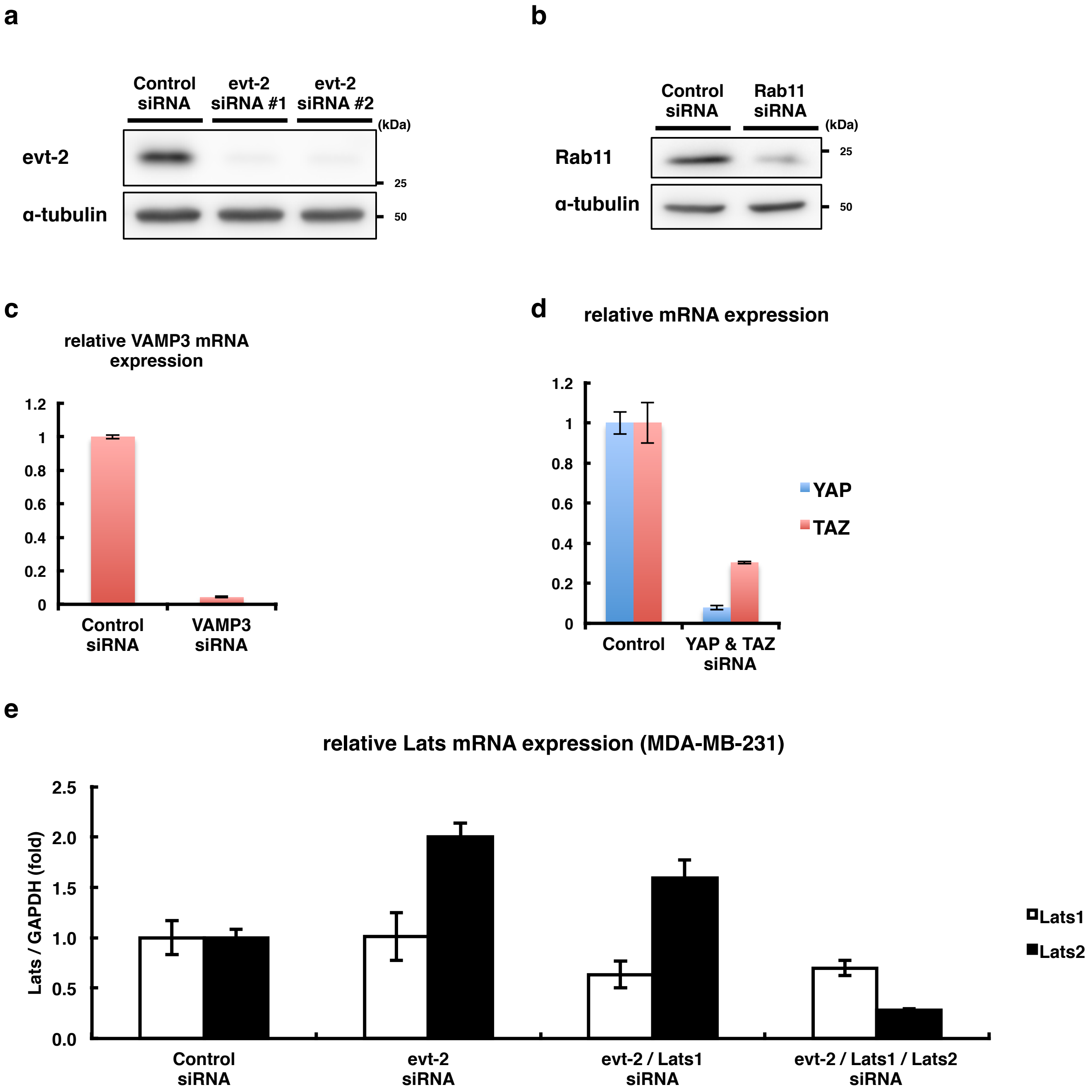

Supplementary Figure 4 Knockdown efficiency of experiments with siRNAs. (**a**) COS-1 cells were treated with control siRNA, evectin-2 siRNA#1, or evectin-2 siRNA#2 for 48 h. Cell lysates were immunoblotted for evectin-2. α-tubulin was used as a loading control. (**b**) COS-1 cells were treated with control or Rab11a siRNA for 48 h. Cell lysates were immunoblotted for Rab11. α-tubulin was used as a loading control. (**c**) qRT-PCR analysis of VAMP3 mRNA in COS-1 cells treated with VAMP3 siRNA for 48 h. GAPDH was used as an internal control. (**d**) qRT-PCR analysis of YAP and TAZ mRNAs in MDA-MB-231 cells treated with YAP and TAZ siRNAs for 48 h. GAPDH was used as an internal control. (**e**) qRT-PCR analysis of Lats1 and Lats2 mRNAs in MDA-MB-231 cells treated with the indicated siRNAs. GAPDH was used as an internal control. Data are mean ± s.d. from two (for **c**) or three (for **d**,**e**) independent experiments.

a peptides identified by MS

ITCH	676 **DLESIDPEFYNSLIWVK** 692
ITCH	808 **QIMWFWQFVK** 817
ITCH	91 **SDVLLGTAALDIYETLK** 107
ITCHI WWP1	654I673 **FIDTGFSLPFYK** 665I684
WWP2	723 **AFLDGFNEVAPLEWLRYFDEK** 743

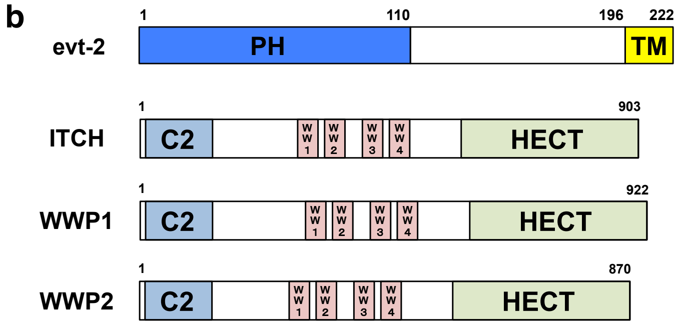

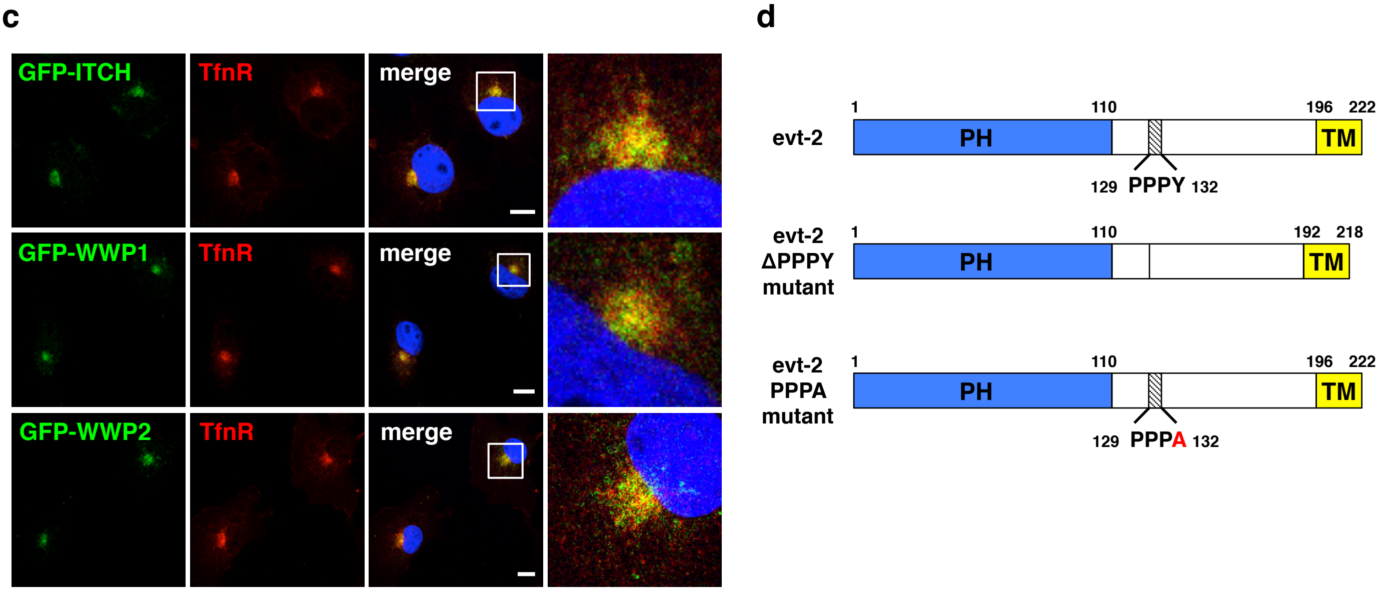

Supplementary Figure 5 evectin-2 binds to Nedd4 E3 ligases. (**a**) Lysates of HEK293T cells that express FLAG-evectin-2 were immunoprecipitated with FLAG-M2 beads. The immunoprecipitates were analyzed by mass spectrometry. Five peptides that corresponded to parts of Nedd4 E3 ligases (Itch, WWP1, and WWP2) were detected. (**b**) Domain architecture of evectin-2 and Nedd4 E3 ligases: PH, pleckstrin homology domain; TM, transmembrane domain; C2, Ca^{2+} /lipid-binding domain; WW1-4, WW domain; HECT, homologous to the E6AP carboxyl terminus domain. (**c**) GFP-tagged proteins were expressed in COS-1 cells. Cells were then fixed, permeabilized, and stained for TfnR. Nuclei were stained with DAPI. Magnified images of the boxed areas around the perinuclear REs are shown in the right column. Scale bars, 10 µm. (**d**) evectin-2 (WT) and two evectin-2 mutants (ΔPPPY and PPPA).

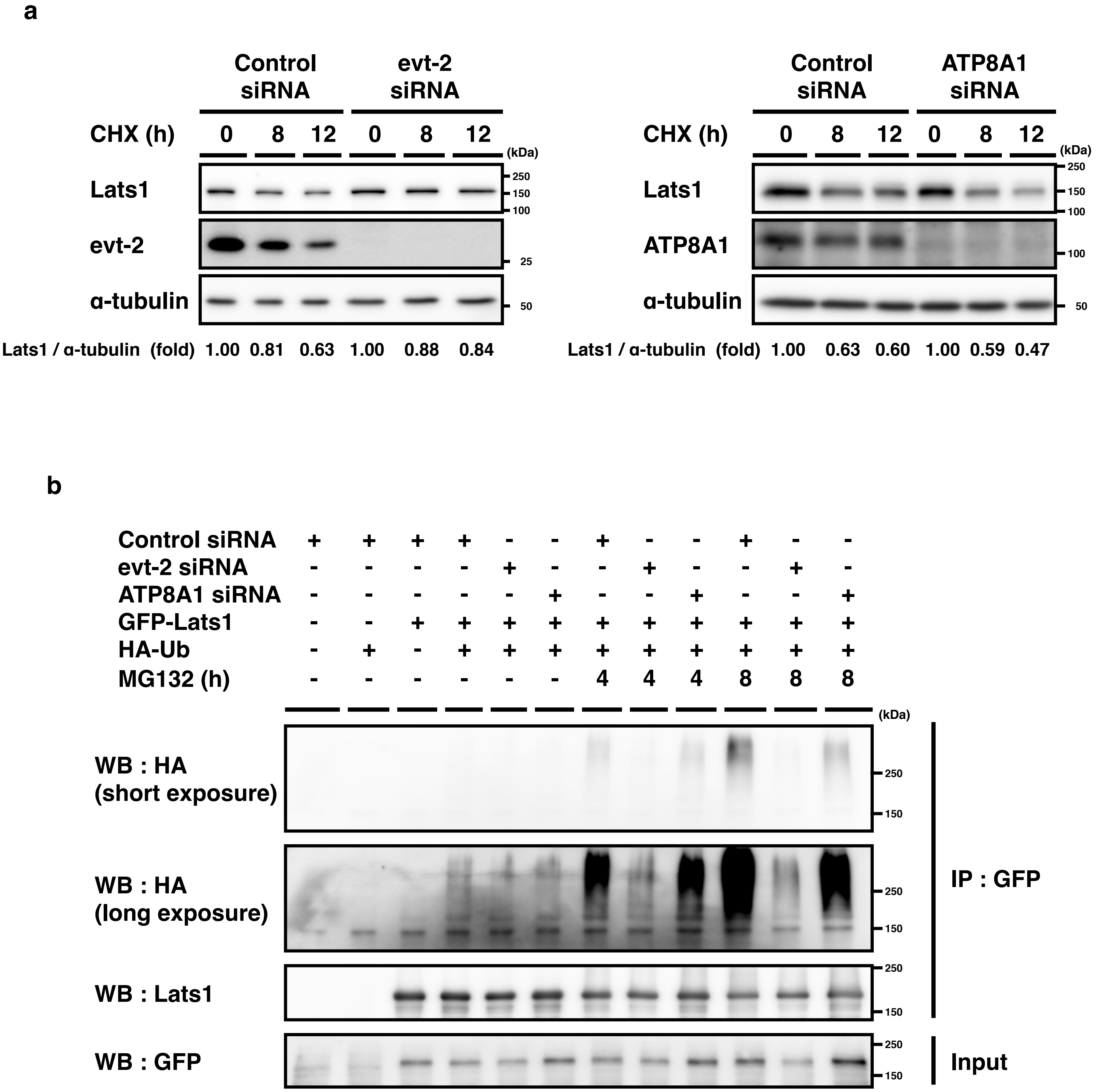

Supplementary Figure 6 Evectin-2 but not ATP8A1 mediates the ubiquitination and degradation of Lats1. (**a**) COS-1 cells were treated with control, evectin-2 siRNA, or ATP8A1 siRNA for 48 h, followed by the incubation with cycloheximide (10 mg mL^{-1}) for the indicated times. Lysates from the cells were immunoblotted for the indicated proteins. α-tubulin was used as a loading control. (**b**) COS-1 cells were treated with control siRNA, evectin-2 siRNA, or ATP8A1 siRNA for 24 h, followed by the transfection of HA-Ubiquitin and GFP-Lats1 for 24 h. The cells were then treated with MG132 (10 μM) for the indicated times. Cell lysates were immunoprecipitated with anti-GFP antibody. The lysates and the immunoprecipitates were then blotted for HA, Lats1, and GFP.

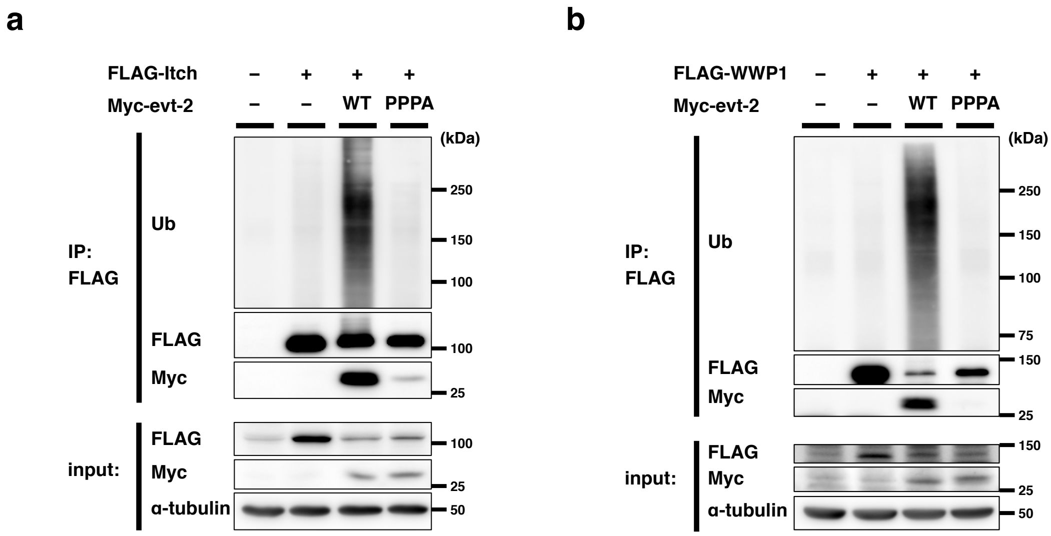

Supplementary Figure 7 Enhanced ubiquitination of Nedd4 E3 ligases with the expression of evectin-2 (WT), but not evectin-2 mutant (PPPA). (**a**,**b**) FLAG-Itch in **a** or FLAG-WWP1 in **b** was co-expressed with Myc-evectin-2 (WT or PPPA mutant) in HEK293T cells for 24 h. Cell lysates with 1% Triton X-100 were immunoprecipitated with anti-FLAG antibody. The immunoprecipitates were then blotted for ubiquitin (Ub), FLAG, or Myc. α-tubulin was used as a loading control.

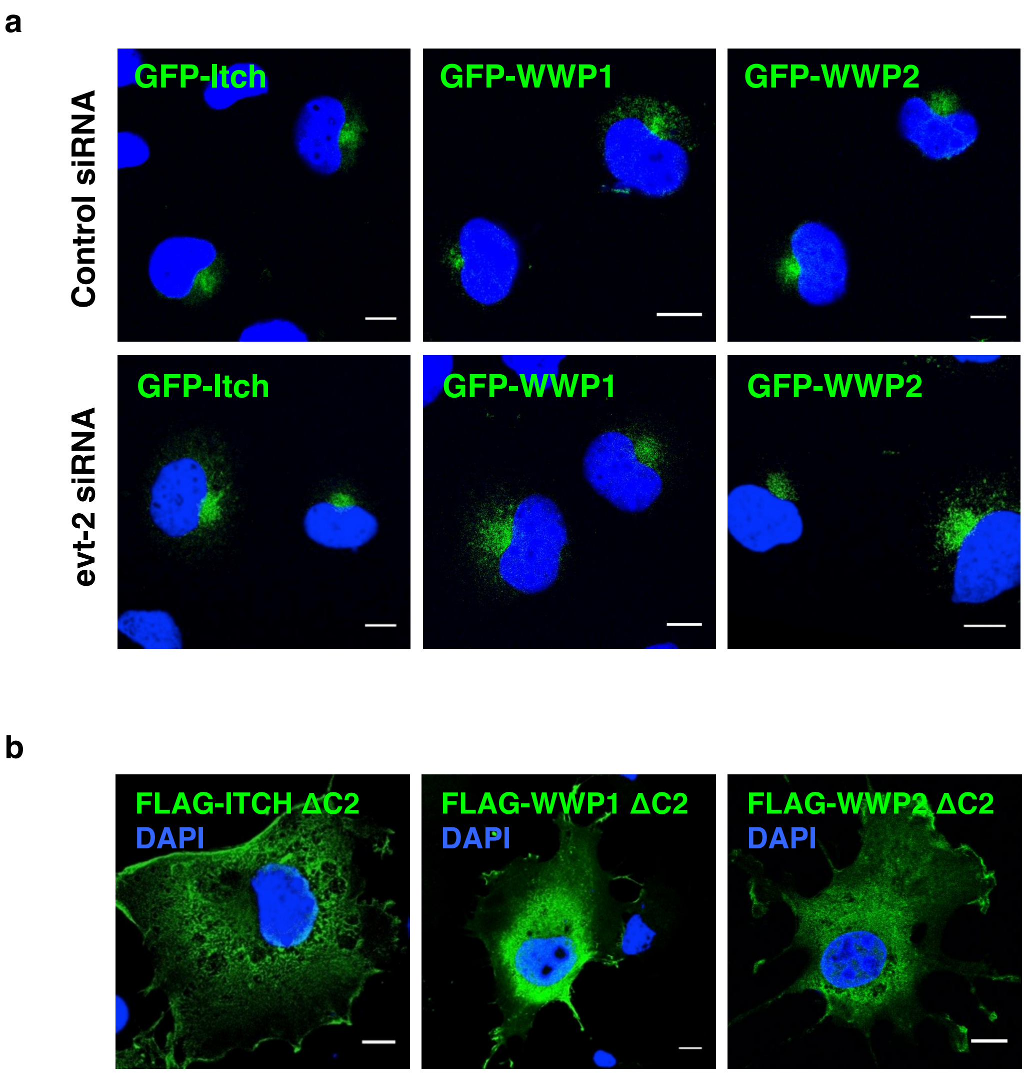

Supplementary Figure 8 (**a**) COS-1 cells that stably express GFP-Itch, GFP-WWP1, or GFP-WWP2 were treated with control or evectin-2 siRNA for 48 h. Cells were then fixed and examined by confocal microscopy. (**b**) FLAG-ITCH that lacks a C2 domain (FLAG-ITCHΔC2), FLAG-WWP1ΔC2, or FLAG-WWP2ΔC2 was expressed in COS-1 cells for 24 h. Nuclei were stained with DAPI. Scale bars, 10 μm.

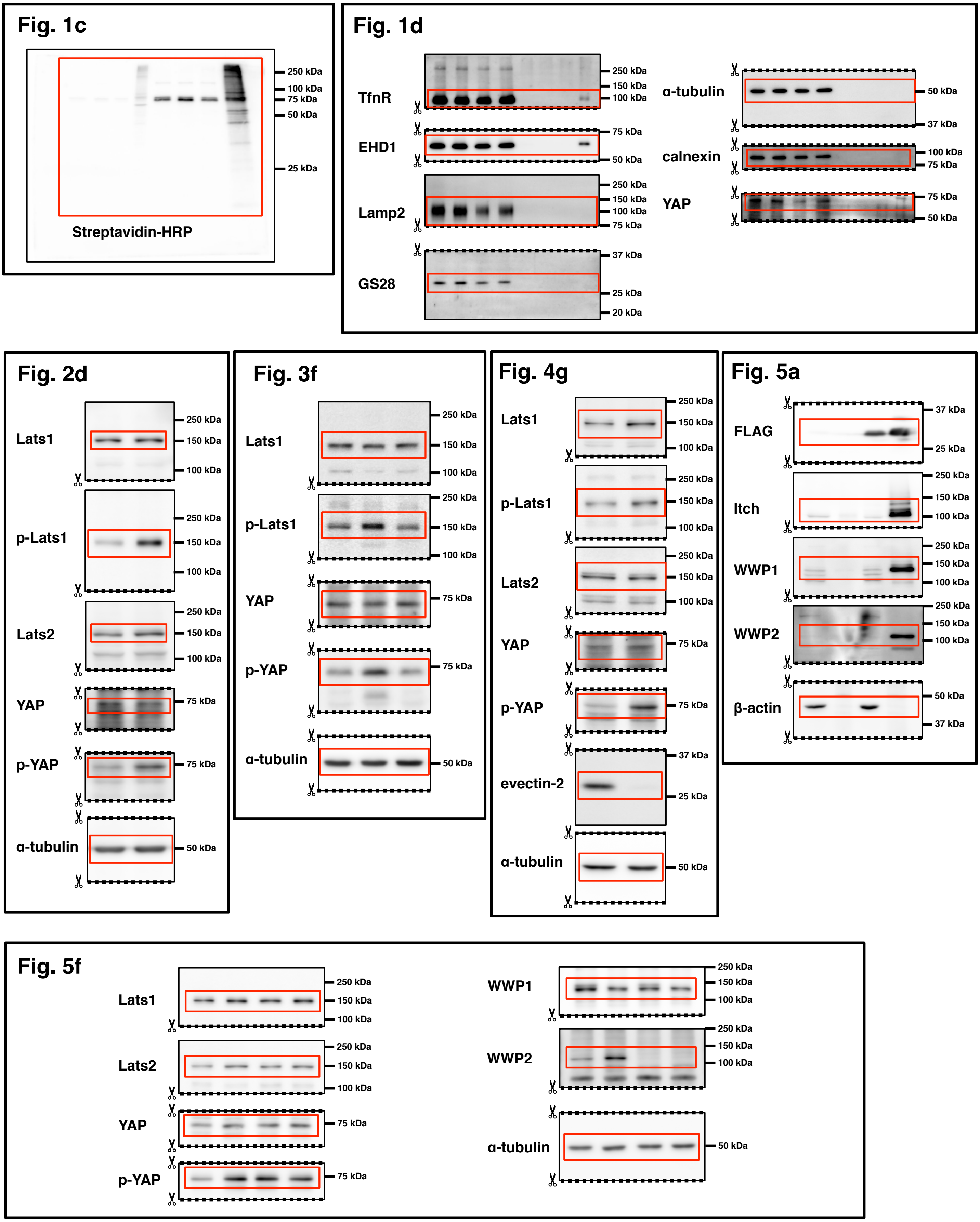

Supplementary Figure 9. Original Western blotting images used in this study.

Fig. 5g

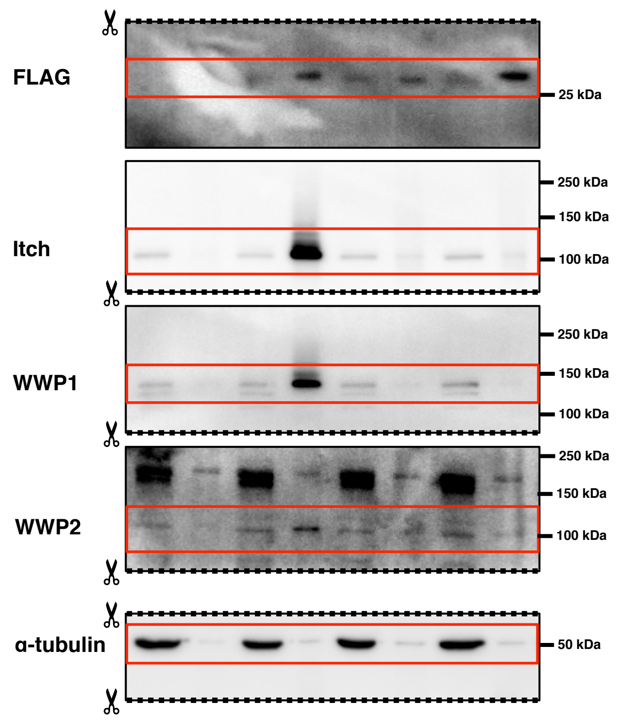

Fig. 5j

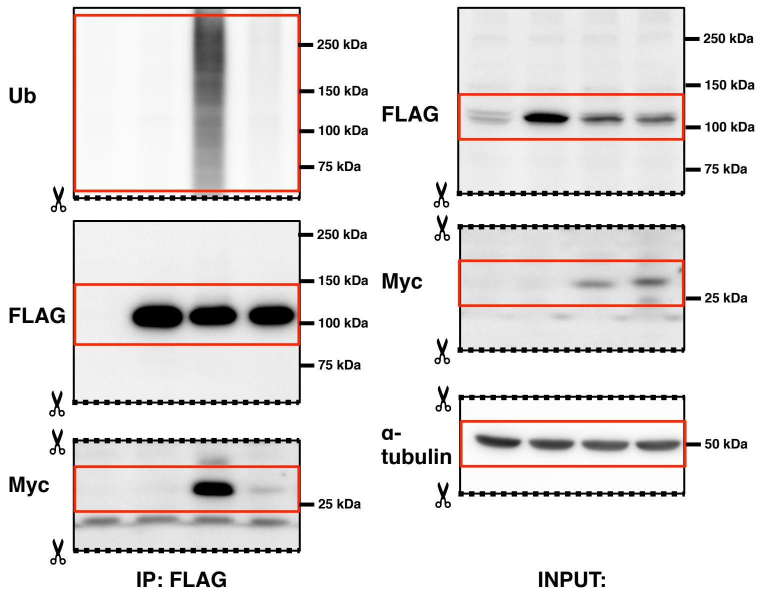

Supplementary Figure 3

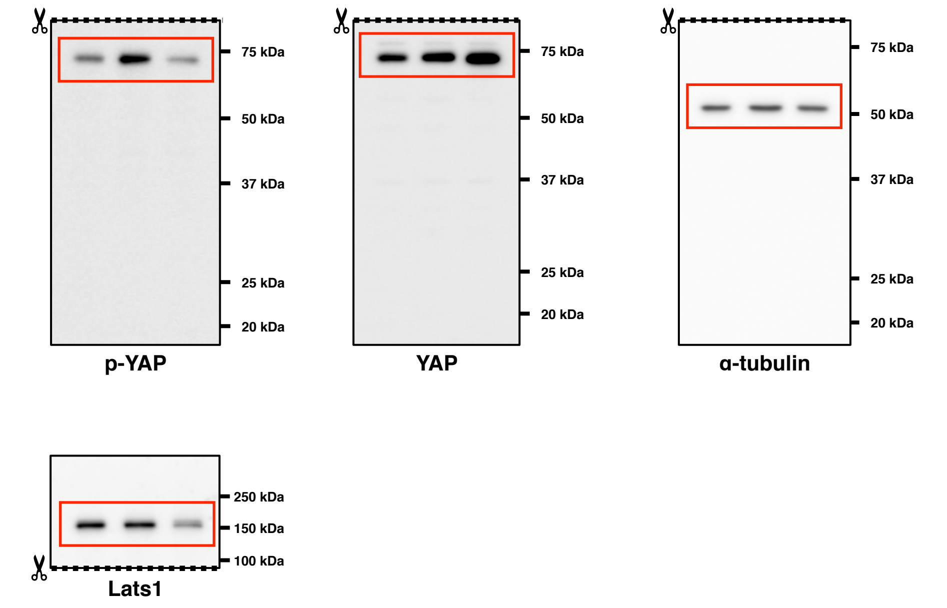

Supplementary Figure 4a

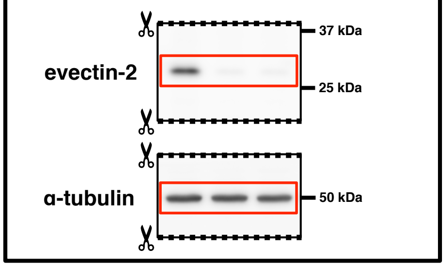

Supplementary Figure 4b

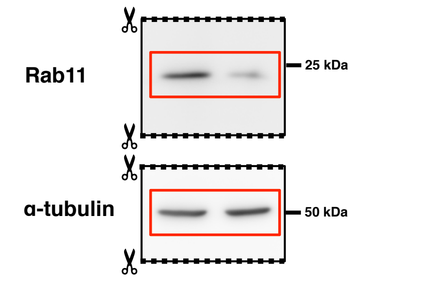

Supplementary Figure 9 (continued). Original Western blotting images used in this study.

Supplementary Figure 6a

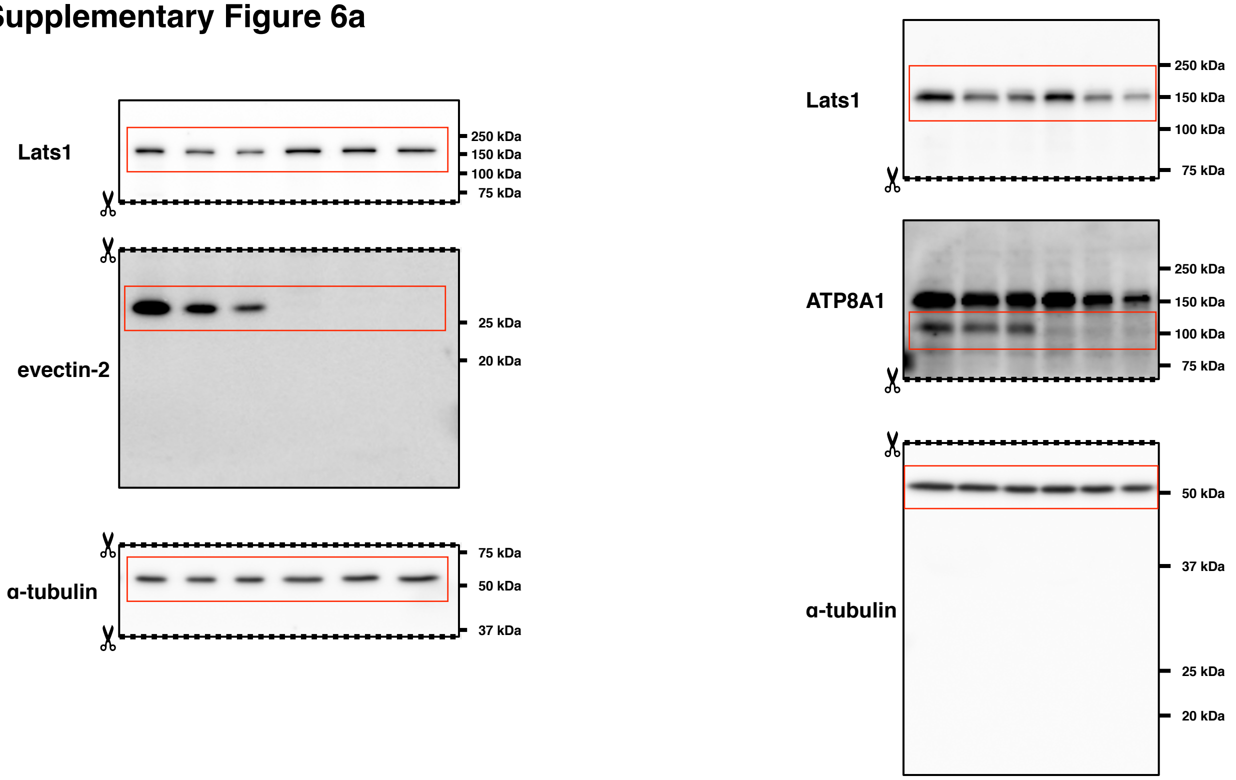

Supplementary Figure 6b

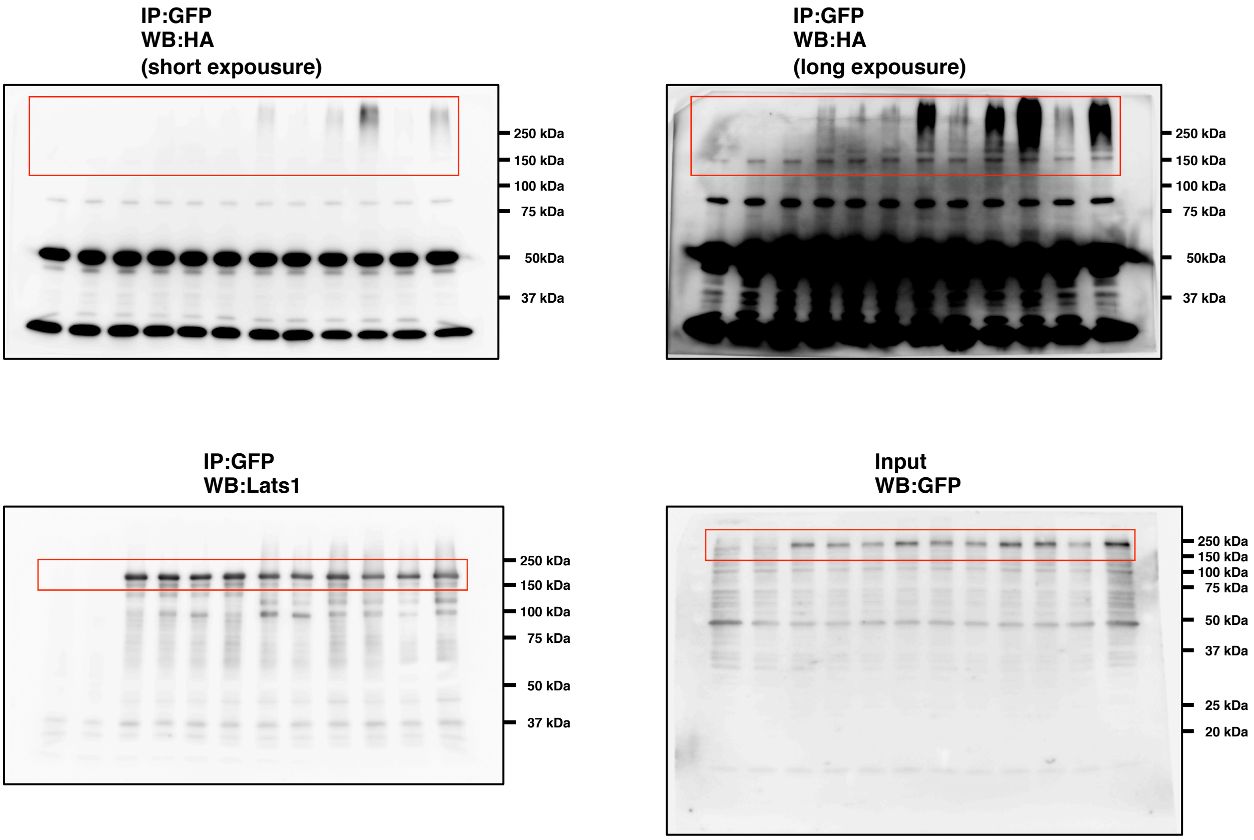

Supplementary Figure 9 (continued). Original Western blotting images used in this study.

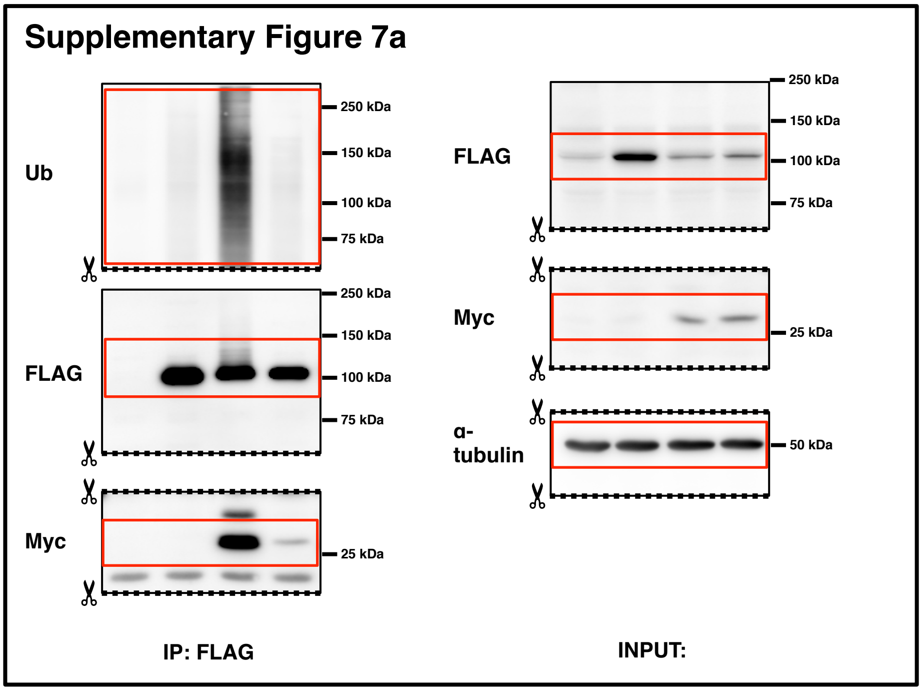

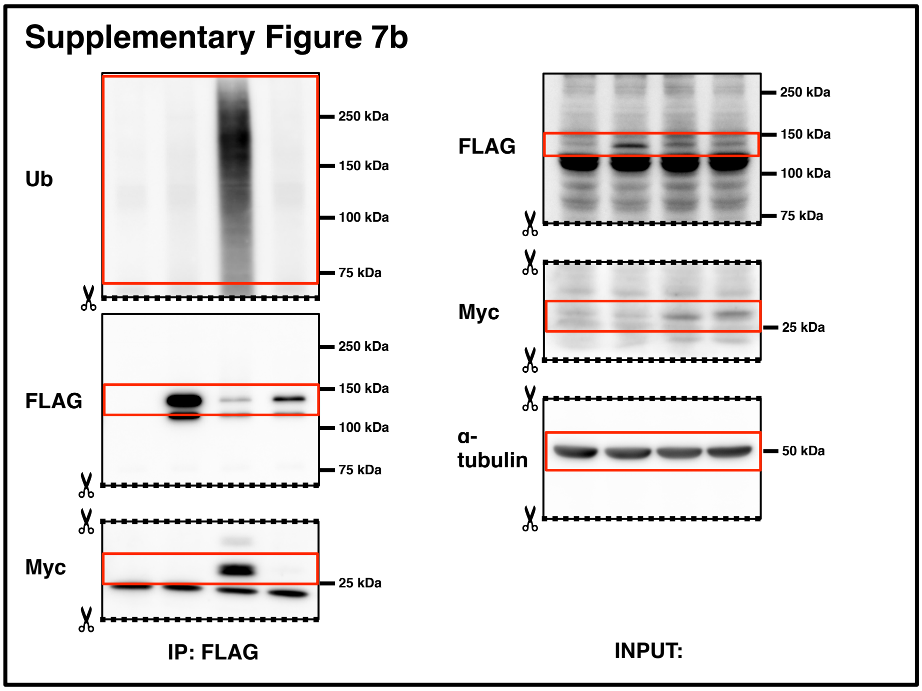

Supplementary Figure 9 (continued). Original Western blotting images used in this study.

Protein name (HUMAN)	Uniprot Entry (HUMAN)	YAP signaling	Ref.	endosome localization
**ANKHD1**	Q8IWZ3	positive	1	
**CD44**	P16070	positive	2	✓
**CDK1**	P06493	positive	3	
**DLG1**	Q12959	negative	4	✓
**ITGB1**	P05556	positive	5	✓
**LATS2**	Q9NRM7	negative	6	
**LLGL1**	Q15334	negative	7	✓
**MAP4K4**	O95819	positive	8	
**MARK2 I MARK3**	Q7KZI7 I P27448	negative	9	
**PAK1**	Q13153	positive	10	
**SKP1**	P63208	negative	11	
**SRC**	P12931	positive	12	✓
**STK38**	Q15208	negative	13	
**YAP1**	P46937	positive	14	the present study
**YES1**	P07947	positive	15	✓
**ZYX**	Q15942	positive	16	✓

1	Machado-Neto et al.	Esp Cell Res	2014	ANKHD1, a novel component of the Hippo signaling pathway, promotes YAP1 activation and cell cycle progression in prostate cancer cells
2	Zhang et al.	Cell Signal	2014	CD44 acts through RhoA to regulate YAP signaling
3	Yang et al.	Cancer Res	2013	CDK1 Phosphorylation of YAP Promotes Mitotic Defects and Cell Motility and Is Essential for Neoplastic Transformation
4	Wu et al.	Hepatology	2016	Zinc Finger Protein 191 Inhibits Hepatocellular Carcinoma Metastasis Through Discs Large 1-Mediated Yes-Associated Protein Inactivation
5	Elbediwy et al.	Development	2016	Integrin signalling regulates YAP/TAZ to control skin homeostasis
6	Zhao et al.	Genes Dev	2007	Inactivation of YAP oncoprotein by the Hippo pathway is involved in cell contact inhibition and tissue growth control
7	Grzeschik et al.	Curr Biol	2010	Lgl, aPKC, and Crumbs Regulate the Salvador/Warts/Hippo Pathway through Two Distinct Mechanisms
8	Zheng et al.	Dev Cell	2015	Identification of Happyhour/MAP4K as Alternative Hpo/Mst-like Kinases in the Hippo Kinase Cascade
9	Mohseni et al.	Nat Cell Biol	2014	A genetic screen identies an LKB1 MARK signalling axis controlling the Hippo YAP pathway
10	Nguyen et al.	Cell Rep	2014	Viral Small T Oncoproteins Transform Cells by Alleviating Hippo-Pathway-Mediated Inhibition of the YAP Proto-oncogene
11	Zhao et al.	Genes Dev	2009	A coordinated phosphorylation by Lats and CK1 regulates YAP stability through SCFβ-TRCP
12	Enomoto et al.	EMBO Rep	2012	Src controls tumorigenesis via JNK-dependent regulation of the Hippo pathway in Drosophila
13	Zhang et al.	Curr Biol	2015	NDR Functions as a Physiological YAP1 Kinase in the Intestinal Epithelium
14	Huang et al.	Cell	2005	The Hippo Signaling Pathway Coordinately Regulates Cell Proliferation and Apoptosis by Inactivating Yorkie, the Drosophila Homolog of YAP
15	Rosenbluh et al.	Cell	2012	b-Catenin-Driven Cancers Require a YAP1 Transcriptional Complex for Survival and Tumorigenesis
16	Ma et al.	Nat Commun	2016	Zyxin-Siah2–Lats2 axis mediates cooperation between Hippo and TGF-β signalling pathways

Supplementary Table 1 Proteins associated with YAP signalling. Among the proteins identified with BirA*-2xPH, proteins that have been reported to be associated with YAP signalling are listed. Proteins that are reported to localize to endosomes are checked (see Supplementary Data 1).

gene name	Forward (5’-3‘)	Reverse (5’-3’)
human CTGF	GCAGAGCCGCCTGTGCATGG	GGTATGTCTTCATGCTGG
human GAPDH	GCCAAGGTCATCCATGACAACT	GAGGGGCCATCCACAGTCTT
human VAMP3	CGCAGCCAAGTTGAAGAGGA	CAGCAGTTTTGAGTTCCGCTG
human YAP	TAGCCCTGCGTAGCCAGTTA	TCTCGAGAGTGATAGGTGCCA
human TAZ	GGCTGGGAGATGACCTTCAC	CTGAGTGGGGTGGTTCTGCT
human Lats1	TGGTCATATTAAATTGACTGAC	CCACATCGACAGCTTGAGGG
human Lats2	TCATCCACCGAGACATCAAGCC	TTGTGAGTCCACCTGAACCCAGTG
Chinese hamster CTGF	GCAGAGTCGCCTCTGCATGG	GGTAGGTCTTCACACTGG
Chinese hamster GAPDH	AGGTCGGTGTGAACGGATTTG	TGTAGACCATGTAGTTGAGGTCA

Supplementary Table 2. primer sequences for qRT PCR
