# Supplementary material for: Endosomal phosphatidylserine is critical for the YAP signalling pathway in proliferating cells
Source: Nat Commun. 2017 Nov 1;8:1246. doi: 10.1038/s41467-017-01255-3 (PMC5665887; doi:10.1038/s41467-017-01255-3)
Supplement: Supplementary file 3 — Description of Additional Supplementary Files [file 41467_2017_1255_MOESM3_ESM.pdf]

Description of Additional Supplementary Files

File Name: Supplementary Data 1

Description: **Proteins identified by MS with BirA*-2xPH.**

sheet 1: iBQ values of the identified proteins by LC-MS/MS. Experiments were performed twice with or without biotin.

sheet 2: 51 proteins showed the ratio of iBQ value [the mean iBQ value (biotin (+)) / the mean iBQ value (biotin (-))] more than 10. 334 proteins were identified only in the presence of biotin and showed the mean iBQ value (biotin (+)) more than 4. Proteins that are reported to localize at endosomes are checked.

sheet 3: information of the peptide sequences identified by LC-MS/MS.
